# Inhibition of Prolyl Oligopeptidase Restores Prohibitin 2 Levels in Psychosis Models: Relationship to Cognitive Deficits in Schizophrenia

**DOI:** 10.3390/ijms24076016

**Published:** 2023-03-23

**Authors:** Èlia Vila, Raquel Pinacho, Roger Prades, Teresa Tarragó, Elena Castro, Eva Munarriz-Cuezva, J. Javier Meana, Ania Eugui-Anta, Mònica Roldan, América Vera-Montecinos, Belén Ramos

**Affiliations:** 1 Parc Sanitari Sant Joan de Déu, Institut de Recerca Sant Joan de Déu, Dr. Antoni Pujadas, 42, 08830 Sant Boi de Llobregat, Spain; 2 Iproteos S.L., Baldiri i Reixac, 10, 08028 Barcelona, Spain; 3 Institute for Research in Biomedicine (IRB Barcelona), Baldiri i Reixac, 10, 08028 Barcelona, Spain; 4 Departamento de Fisiología y Farmacología, Universidad de Cantabria, Avda. Cardenal Herrera Oria s/n, 39011 Santander, Spain; 5 Centro de Investigación Biomédica en Red de Salud Mental, CIBERSAM (Biomedical Network Research Center of Mental Health), Institute of Health Carlos III, 28029 Madrid, Spain; 6 Department of Pharmacology, University of the Basque Country UPV/EHU, 48940 Leioa, Spain; 7 Biocruces Bizkaia Health Research Institute, 48903 Barakaldo, Spain; 8 Unitat de Microscòpia Confocal i Imatge Cel·lular, Servei de Medicina Genètica i Molecular, Institut Pediàtric de Malaties Rares (IPER), Hospital Sant Joan de Déu, 08950 Esplugues de Llobregat, Spain; 9 Departament de Bioquímica i Biologia Molecular, Facultat de Medicina, Universitat Autònoma de Barcelona, Bellaterra, 08193 Barcelona, Spain; 10 Faculty of Medicine, University of Vic-Central University of Catalonia, 08500 Vic, Spain

**Keywords:** schizophrenia, PHB2, cognitive deficits, postmortem, DLPFC, prolyl oligopeptidase, dizocilpine

## Abstract

Cognitive impairment represents one of the core features of schizophrenia. Prolyl Oligopeptidase (POP) inhibition is an emerging strategy for compensating cognitive deficits in hypoglutamatergic states such as schizophrenia, although little is known about how POP inhibitors exert their pharmacological activity. The mitochondrial and nuclear protein Prohibitin 2 (PHB2) could be dysregulated in schizophrenia. However, altered PHB2 levels in schizophrenia linked to N-methyl-D-aspartate receptor (NMDAR) activity and cognitive deficits are still unknown. To shed light on this, we measured the PHB2 levels by immunoblot in a postmortem dorsolateral prefrontal cortex (DLPFC) of schizophrenia subjects, in the frontal pole of mice treated with the NMDAR antagonists phencyclidine and dizocilpine, and in rat cortical astrocytes and neurons treated with dizocilpine. Mice and cells were treated in combination with the POP inhibitor IPR19. The PHB2 levels were also analyzed by immunocytochemistry in rat neurons. The PHB2 levels increased in DLPFC in cases of chronic schizophrenia and were associated with cognitive impairments. NMDAR antagonists increased PHB2 levels in the frontal pole of mice and in rat astrocytes and neurons. High levels of PHB2 were found in the nucleus and cytoplasm of neurons upon NMDAR inhibition. IPR19 restored PHB2 levels in the acute NMDAR inhibition. These results show that IPR19 restores the upregulation of PHB2 in an acute NMDAR hypoactivity stage suggesting that the modulation of PHB2 could compensate NMDAR-dependent cognitive impairments in schizophrenia.

## 1. Introduction

Schizophrenia (SZ) is a complex mental disorder in which genetic and environmental risk factors interact during development, leading to synaptic and plasticity disruption in different brain areas [1]. Several hypotheses have been proposed to explain the underlying causes of SZ. The neurodevelopmental hypothesis of SZ suggests that disruptions in the brain could be, in part, a consequence of events occurring early in the development that account for the later manifestation of symptoms of SZ [2]. According to the neurodevelopmental hypothesis, altered specific neural circuits and an excessive elimination of synapses could lead to a loss of plasticity later in adolescence [3,4]. Several neurotransmitter systems, including dopaminergic, glutamic acid decarboxylase 67, and glutamatergic, among others, have been implicated in the pathophysiology of SZ [5]. On the basis of preclinical and clinical research, the inhibition of the non-competitive N-methyl-D-aspartate (NMDA) receptor antagonist-induced symptoms observed in SZ supports the implication of the dysregulation of glutamatergic neurotransmission in the pathophysiology of SZ [6]. Indeed, multiple sources of evidence support the role of the glutamatergic system in cognitive deficits in SZ [7,8]. 

Symptoms of SZ are diverse but are typically divided into positive, negative, and cognitive symptoms. Neurocognitive dysfunction has been postulated to play a central role in many psychiatric disorders, and the deficits in several cognitive domains in people with SZ have been widely investigated [9,10]. Since the efficacy of the current antipsychotic drugs on cognition is limited, the cognitive dysfunction in SZ is the current focus of attention for the research of therapeutic and pharmacological interventions [11,12,13]. Several studies suggest that neuropeptides may contribute to the pathophysiology of SZ [14,15]. Changes in Prolyl Oligopeptidase (POP, aka Prolyl Endopeptidase (PREP) have been described in SZ and are linked to glutamatergic neurotransmission in the brain [16,17,18]. Indeed, POP inhibitors have been described as an emerging approach for disorders related to cognitive impairments, as in Alzheimer’s and Parkinson’s diseases [19,20]. Specifically, the POP inhibitor IPR19 (ACT-02) has been suggested as a strategy to ameliorate cognitive deficits in SZ [21,22]. However, the underlying mechanism of action of POP inhibitors are still unknown. 

Structural, functional, and connectivity disturbances have been reported in the prefrontal cortex (PFC) in SZ [23,24,25], and an altered executive function has been associated with prefrontal cortex activity in SZ patients [26,27,28]. Previous research using a proteomic approach has suggested altered levels of Prohibitin 2 (PHB2) in the dorsolateral prefrontal cortex of SZ subjects [29]. The Prohibitin (PHB) complex comprises two highly homologous subunits, PHB1 and PHB2, which are ubiquitously expressed [30]. PHB has been associated with several processes, including mitochondrial membrane degradation, the stabilization of the mitochondrial genome, the regulation and assembly of the oxidative phosphorylation system activity, and mitochondrial apoptosis [31,32,33,34]. More recently, PHB2 has been described as a mitophagy receptor that is involved in targeting mitochondria for autophagic degradation [35,36,37]. In the nucleus, prohibitins have a role as transcriptional co-regulators acting independently from each other as PHB1 and PHB2 [38]. PHB2 was identified as a repressor of the nuclear estrogen receptor [39] and it inhibits the transcriptional activity of other target genes [38]. However, the possible dysregulation of PHB2 in schizophrenia and its possible contribution to the cognitive symptoms observed in the pathology is still unknown.

We aimed to investigate the possible alteration in PHB2 levels in DLPFC tissue from chronic SZ subjects and its association with cognitive deficits. Moreover, we aimed to investigate the modulation of PHB2 levels by NMDAR activity in animal and cellular models. Although there is no previous link between the cognitive enhancer IPR19 and PHB2, we also explored the possible modulation of PHB2 by IPR19.

## 2. Results

### 2.1. Analysis of PHB2 Levels in Postmortem Dorsolateral Prefrontal Cortex in Schizophrenia

**Figure 1:**
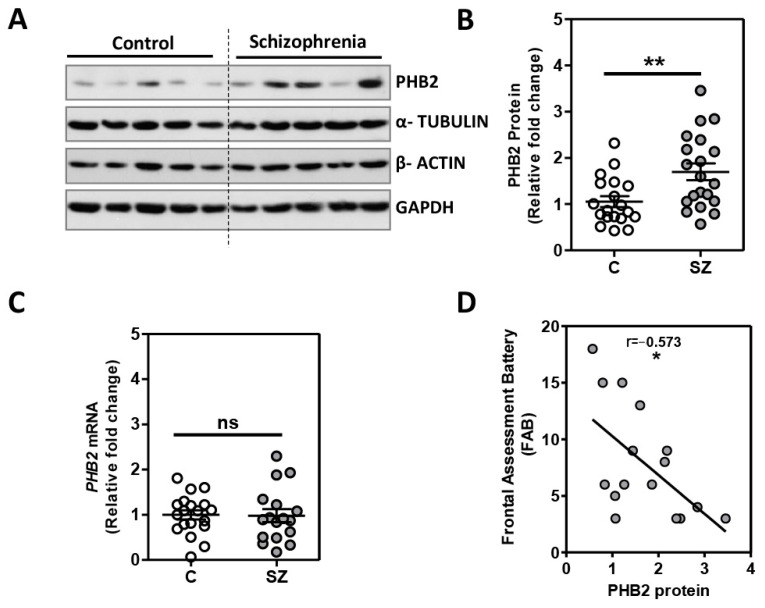
Increased protein levels, but not mRNA levels, of PHB2 in dorsolateral prefrontal cortex of chronic schizophrenia associated with altered executive function: (**A**) Representative immunoblot images for PHB2 and the geometrical mean of the housekeeping proteins (HK): α-Tubulin, β-Actin, and GAPDH from the prefrontal cortex of schizophrenia patients (SZ = 20) and unaffected control individuals (C = 20). (**B**) Data represents the mean + standard error of the mean for each group for PHB2 protein levels. Protein levels of PHB2 were normalized to the geometrical mean of HK protein values and to a reference control sample (Figure S1). One outlier for PHB2 in the control group was detected and therefore excluded from the analysis. (**C**) PHB2 mRNA levels from the prefrontal cortex of schizophrenia patients (SZ = 17) and unaffected control individuals (C = 19) determined by RT-qPCR and normalized to beta glucuronidase (GUSB) levels and a control reference sample (Figure S1). (**D**) Association between PHB2 protein levels from the prefrontal cortex of a subgroup of schizophrenia patients (SZ = 16) and Frontal Assessment Battery (FAB) scores. (* *p* < 0.05; ** *p* < 0.01; ns, not significant).

For the analysis of PHB2 in DLPFC, we used a cohort of elderly SZ patients (n = 20) with a long duration of the illness and matched with unaffected individuals (n = 20). No differences were observed between both groups in any demographic- or tissue-related variables (Table 1). We found that the PHB2 protein levels were significantly increased in SZ (*p* = 0.0051) (t = 2.974, df = 37; Mean ± SEM: control (C) = 1.05 + 0.12; SZ = 1.70 + 0.18) (Figure 1A,B). No differences in the housekeeping protein levels were observed between groups (Figure S1A). We further analyzed PHB2 gene expression in a subgroup of SZ patients (n = 17) and matched with unaffected controls (n = 19). We did not find any significant difference in the sociodemographic-, clinical-, and tissue-related features between these subgroups of patients and the unaffected controls (Table S1). No differences in housekeeping gene expression were observed between the groups (Figure S1B). No differences between SZ subjects and unaffected individuals were observed in PHB2 expression levels (t = 0.099, df = 34; Mean ± SEM: control (C) = 1.00 + 0.10; SZ = 0.98 + 0.14]) (Figure 1C). An increase in PHB2 protein levels was also detected in SZ in these subgroups (n = 15, *p* = 0.018) (t = 2.498, df = 33; Mean ± SEM: control (C) = 1.09 + 0.15; SZ = 1.68 + 0.22) (Figure S2). We also analyzed the possible association of other variables in the study (age, postmortem delay, pH, and RIN) with PHB2 protein levels in our cohort. The analysis revealed no association between these potential confounding factors and PHB2 protein levels (Table S2).

Further, we analyzed the possible association between PHB2 protein levels and neurocognitive deficits assessed by the Frontal Assessment Battery (FAB) scores (n = 16). We found a significant inverse association with the FAB scores and PHB2 protein levels (r = −0.573; *p* = 0.020) (Figure 1D). We further assessed the possible influence of years of education on PHB2 levels. No difference in the PHB2 protein levels were found between patients with more or less than 6 years of education (*p* = 0.147) (t = 1.56, df = 11; Mean ± SEM: <6 years of education = 1.79 + 0.34 (n = 7); >6 years of education = 1.19 + 0.12 (n = 6)). No differences in FAB scores were found between patients with more or less than 6 years of education (*p* = 0.326) (t = 1.03, df = 11; Mean ± SEM: <6 years of education = 7 + 2.00 (n = 7); >6 years of education = 10 + 2.11 (n = 6)).

We also analyzed the possible association between PHB2 and the age of onset, daily antipsychotic dose, and duration of the illness in the SZ group. The analysis revealed no association between these potential confounding factors and PHB2 protein levels (Table S2). To match the clinical condition of antipsychotic medications, subchronic treatments with atypical antipsychotic clozapine and the typical antipsychotic haloperidol in animals were used. The possible effect on PHB2 protein levels in rats treated daily for 21 days (n = 6/group) was analyzed. No differences in PHB2 protein levels were observed upon treatment with these antipsychotics (ANOVA: F[2,15] = 1.494; *p* = 0.256) (Figure S3).

### 2.2. Modulation of PHB2 Protein Levels by NMDA Receptor Activity and the Prolyl Oligopeptidase Inhibitor IPR19 in Rodents

**Figure 2:**
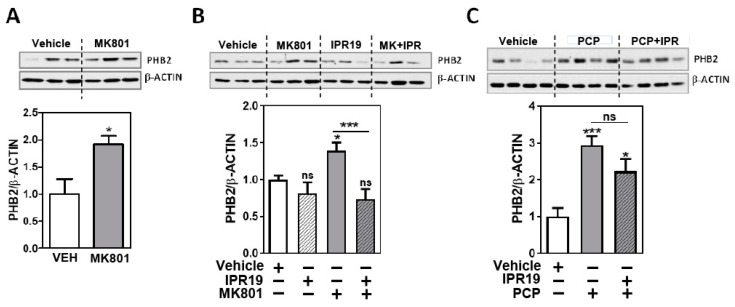
PHB2 protein levels were regulated by a POP inhibitor (IPR19) in the frontal cortex of mice acutely treated with MK801: Representative immunoblot images for PHB2 and β-actin in untreated and treated animals are shown. Each bar represents the mean and the standard error of the mean for each group for PHB2 normalized to β-actin. (**A**,**B**) PHB2 protein levels from the frontal cortex of 8-week-old C57Bl/6 J mice acutely treated (35 min) with vehicle (VEH) or the N-methyl-D-aspartate receptor antagonist MK801 (0.2 mg/kg), (**B**) and with the prolyl oligopeptidase inhibitor IPR19 (5 mg/kg), with and without MK801 (**B**) (n = 6, per group). (**C**) PHB2 protein levels from the frontal cortex of 8 to 10-week-old C57Bl/6 J mice subchronically treated (10 days) with vehicle (VEH; n = 5), phencyclidine (10 mg/kg PCP; n = 8), or PCP with IPR19 (n=8). In both experiments (**B**,**C**), the same IPR19 concentration and exposure times were used (5 mg/kg IPR19) ). Statistical analysis was performed using ANOVA followed by post hoc comparison between untreated condition and different treatments and the indicated comparison with MK801 or PCP group. (* *p* < 0.05, *** *p* < 0.001; ns, not significant).

To investigate whether the PHB2 protein levels could be altered in the early stages of the disease, we determined the PHB2 protein levels in a mouse model of psychosis. Mice were treated with an acute dose of MK801 (n = 6) or a vehicle (n = 6). We observed a significant increase in the PHB2 protein levels in the frontal pole of mice treated with MK801 (*p* = 0.017) (Figure 2A).

Since we observed a negative correlation of the PHB2 protein levels and cognitive deficits (Figure 1D), we further characterized the protein levels of PHB2 in this animal model in combination with the prolyl oligopeptidase inhibitor IPR19 for 35 min (n = 6/group), which has been reported to ameliorate cognitive deficits in preclinical models related to SZ. IPR19 alone did not alter the PHB2 protein levels (*p* = 0.282). IPR19 restored the increased levels induced by MK801 (Figure 2B, ANOVA: F[3,20] = 6.351; *p* = 0.0034).

We further determined the PHB2 protein levels in a subchronic model of SZ mice treated with phencyclidine (PCP) in combination with IPR19 (Figure 2C). We also observed a significant increase in the PHB2 protein levels in animals treated with PCP (n = 8) compared with those treated with the vehicle (n = 5). However, the PHB2 protein levels were not significantly altered under both the IPR19 and PCP treatments (Figure 2C, ANOVA: F[2,18] = 9.639 *p* = 0.0014).

### 2.3. Modulation of PHB2 Protein Levels by NMDA Receptor Activity and the Prolyl Oligopeptidase Inhibitor IPR19 in Rat Cortical Astrocytes and Neurons

#### 2.3.1. Acute Treatments

**Figure 3:**
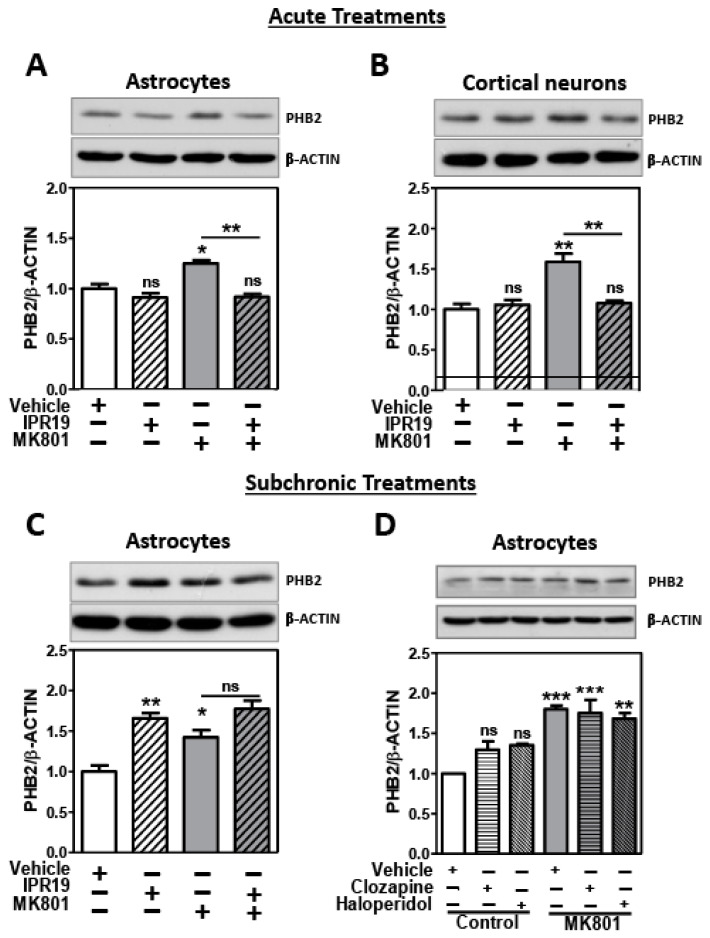
PHB2 protein levels were regulated by the POP inhibitor (IPR19) in both cortical neurons and astroglial-enriched cultures acutely treated with MK-801: Representative immunoblot images for PHB2 and β-actin in untreated and treated cells are shown. Each bar represents the mean and the standard error of the mean for each group for PHB2 normalized to β-actin. PHB2 protein levels from astroglial-enriched cultures (**A**) and cortical frontal neurons (**B**) acutely treated with vehicle (DMSO), N-methyl-D-aspartate receptor antagonist MK801 (10 µM MK801), prolyl oligopeptidase inhibitor IPR19 (50 µM IPR19), or the combination of both inhibitors (MK801 and IPR19) for 35 min. (**C**) PHB2 protein levels from astroglial-enriched cultures subchronically treated with DMSO, 50 µM MK801, 50 µM IPR19, or the combination of both inhibitors (MK801 and IPR19) during 72 h. (**D**) Astroglial-enriched cultures cultured and treated with MK801 or vehicle (DMSO) together with 10 µM clozapine or 1 µM haloperidol for 72 h. Statistical analysis was performed using one-way (**A**–**C**) or two-way (**D**) ANOVA followed by post hoc comparison between untreated condition and different treatments and the indicated comparison with MK801 group. (* *p* < 0.05; ** *p* < 0.01; *** *p* < 0.001; ns, not significant).

We analyzed the protein levels of PHB2 in cortical neurons treated with 10 µM MK801 or combined with 50 µM IPR19 for 35 min. We also observed a significant increase in the protein levels of PHB2 with the MK801 treatment. IPR19 treatment in the presence of MK801 restored the PHB2 levels to control conditions (ANOVA: F[3,8] = 11.68; *p* = 0.0027) (Figure 3A). These results reproduce those observed in the tissue in the acute model of psychosis (Figure 2B). In addition, we analyzed the protein levels of PHB2 in cortical astroglial-enriched cultures with the same conditions as in astrocytes. We observed the same results in both cell types; an increase in PHB2 protein levels when NMDAR were blocked with MK801, and a restoration of PHB2 protein levels upon IPR19 treatment (ANOVA: F[3,8] = 17.3; *p* = 0.0007) (Figure 3B).

#### 2.3.2. Subchronic Treatments

Astrocytes were treated for 72 h with 50 µM IPR19, 50 µM MK801 NMDA antagonist, or the combination of both inhibitors. The PHB2 protein levels were increased with MK801 treatment but IPR19 could not counteract the effect of MK801 on the PHB2 protein levels (ANOVA: F[3,8] = 16.45; *p* = 0.0009) (Figure 3C). Since IPR19 did not modulate PHB2 in subchronic treatments, we further investigated the possibility that subchronic antipsychotic treatments could modulate PHB2 protein levels alone and under NMDAR blockade. We analyzed the effect of atypical antipsychotic clozapine, and the typical antipsychotic haloperidol on astroglial-enriched cultures subchronically treated with a 50 µM MK801 NMDA antagonist for 72 h. A statistically significant interaction was found between the effects of MK801 and antipsychotic treatments (Two way ANOVA: F[2,12] = 3.911, *p* = 0.047). There was no statistically significant difference in the PHB2 protein levels between the vehicle and clozapine group (*p* = 0.479) and the vehicle and haloperidol group (*p* = 0.199). Significant differences in the PHB2 protein levels were found between the control and MK801 (*p* = 0.0004), clozapine and MK801 (*p* = 0.0007), and haloperidol and MK801 (*p* = 0.0018) groups.

### 2.4. Characterization of PHB2 in Cortical Neuron Cultures Treated with NMDA Receptor Antagonist and the Inhibitor IPR19

**Figure 4:**
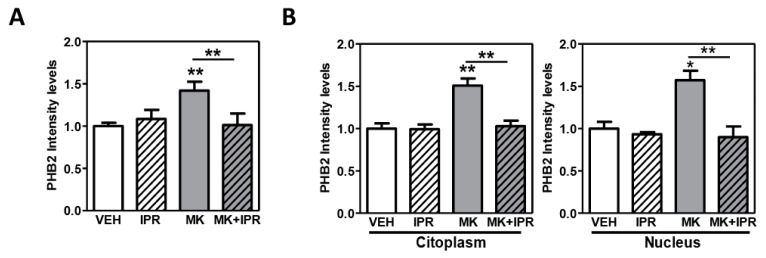
Immunocytochemistry of PHB2 in cortical neurons: (**A**) Each bar represents the mean and the standard error of the mean for each group for PHB2 protein intensity levels referred to the number of nuclei and the control group of three independent experiments. (**B**) Each bar represents the mean and the standard error of the mean for the nuclear and cytoplasm PHB2 protein intensities in all treatment conditions. Levels referred to the number of nuclei and the control group of three independent experiments. (* *p* < 0.05, ** *p* < 0.01).

Since the PHB2 changes upon the inhibition of NMDA receptor activity and its modulation by IPR19 were more prominent in cortical neuron cultures compared to astrocytes (Figure 3B), we aimed to investigate the subcellular localization of PHB2 upon treatment with these inhibitors. We first confirmed the modulation of PHB2 protein levels in cortical neurons treated with MK801 and IPR19 using confocal microscopy. We found that the immunodetection of PHB2 was significantly increased in cells treated with MK801. In the presence of MK801, IPR19 treatment restored PHB2 levels similar to those observed in the control condition (ANOVA: F[3,8] = 10.90; *p* = 0.0034) (Figure 4A). These results reproduce those previously observed by immunoblotting (Figure 3B). We analyzed the fluorescence intensity of PHB2 in the cytoplasm and in the nucleus of individual cells. We observed an increase in the PHB2 protein levels in both cytoplasm (ANOVA: F[3,20] = 13.60; *p* = 0.0017) and nucleus (ANOVA: F[3,8] = 11.32; *p* = 0.0030) under MK801 treatment. A significant decrease in PHB2 levels was found in both the subcellular compartments when cells were also treated with IPR19 (Figure 4B and Figure S4).

To investigate the subcellular localization of PHB2 in cortical neurons under MK801 treatment, we used a double staining with DAPI and TOMM20, as nuclear and mitochondria markers, respectively. The TOMM20 levels did not differ between the control and MK801 conditions (*p* = 0.435). Merge images showed that PHB2 co-localizes with TOMM20 in cortical neurons (Figure S5). To determine the co-localization of PHB2 and TOMM20, we analyzed the overlay correlation (Pearson) in treated and untreated neurons. Our analysis shows that PHB2 and TOMM20 are highly correlated in both the untreated (r = 0.777) and MK801 treated (r = 0.814) neurons, suggesting that the mitochondria is the main subcellular localization of PHB2 and the inhibition of the NMDA receptor activity did not alter the localization of PHB2 in this organelle.

## 3. Discussion

Our study shows that the inhibition of prolyl oligopeptidase restores the upregulation of PHB2 protein levels in the acute NMDAR hypoactivity stage in mice and cellular models of SZ. In SZ subjects, here we also report an increase in the PHB2 levels in the dorsolateral prefrontal cortex which inversely correlates with cognitive performance. These results suggest that the modulation of PHB2 could compensate for the NMDAR-dependent cognitive impairments in SZ.

Our data supports an upregulation of the PHB2 protein levels in the DLPFC in SZ. These results are consistent with our previous findings using proteomic approaches published in Pinacho et al., 2016 [29]. Another proteomic study suggested increased PHB2 levels in the anterior cingulate cortex in SZ [40], but, to the best of our knowledge, this is the first time that an increase in the PHB2 protein levels is reported in DLPFC gray matter in individuals with SZ. Here, we observe an increase in the PHB2 protein levels but not in the PHB2 mRNA expression levels in a postmortem brain in SZ, suggesting that post-translational mechanisms could contribute to the differences observed in PHB2 in this disorder. PHBs are regulated through some post-translational modifications that might control their protein stability in this pathological context [41]. However, no studies have explored this possibility yet.

PHB2 was also increased in animal models of psychosis by blocking NMDAR and in cells treated with an inhibitor of this type of receptor. Several studies suggest that there is a hypofunction of the NMDAR in SZ, which contribute to many of the pathological brain processes that occur in the disease process [42,43]. In glutamatergic models, the administration of NMDAR antagonists leads to neurochemical, morphological, and schizophrenia-like behavioral features similar to the ones observed in SZ [44]. Thus, these results suggest that the PHB2 protein levels may participate in the hypoglutamatergy linked to SZ in the first steps of the disease and in the chronic stages of the pathology. However, it is not clear how an increase in the levels of the PHB2 protein could impact glutamate signaling. We also observed that PHB2 protein levels increased in neurons and astrocytes under the acute and subacute NMDAR antagonist MK-801 treatment. These results are in agreement with the previous findings, described in this study in animal models, suggesting that PHB2 may be deregulated in both cell types in the brain in the initial stages of the pathology. Patients with SZ show progressive disturbances in astrocytes leading to an activation of glia, which has an impact on glutamate release and dysregulated synaptic transmission [45,46,47]. Astrocytes are responsible for the conversion of glutamate to glutamine, and changes in the glutamate/glutamine cycle have an impact in the energy exchange between neurons and astrocytes in SZ [48]. Moreover, it has been described that glutamate transmission strongly depends on the mitochondrial function [49] and postmortem evidence suggests progressive disturbances of the astrocyte function due to mitochondria deficits in SZ [50]. Since PHB2 exerts multiple roles in mitochondria dynamics, our results open the possibility that PHB2 could be participating in the altered metabolic state described in this disorder. However, in our study we do not have any evidence to clarify how the cell-type-specific regulation of PHB2 takes place under the NMDAR blockade in the frontal pole in SZ.

The association between DLPFC and cognitive deficits seen in SZ is well established [51,52]. In this work, we observed that PHB2 protein levels changed in relation with the FAB scores in the DLPFC of SZ patients, which highlight that people with SZ with higher levels of PHB2 may have poorer cognitive performances. Dysfunctions in glutamatergic neurotransmission systems and mitochondrial dysfunctions have been implicated in the emergence of these symptoms in SZ [53,54,55]; however, to the best of our knowledge, no studies are available for PHB2 protein in this context and more studies are needed to elucidate by which mechanisms PHB2 could contribute to the cognitive impairments observed in SZ. Recently, several POP inhibitors have been developed for the treatment of CNS disorders and it has been suggested that the POP inhibitor, IPR19, improves cognitive performance and it might have therapeutic potential for the treatment of the cognitive deficits associated with SZ [21,22]. In this study, we observed that the PHB2 protein levels were restored by treatment with the cognitive enhancer IPR19 in acute, but not in subchronic, animal and cell MK801 models. Together, these results highlight that an increase in PHB2 protein levels could contribute to the cognitive impairments described in SZ patients and could be modulated in the early stages of the disease by the cognitive enhancer IPR19. However, in our study, we do not have any explanation as to why IPR19 is able to restore PHB2 protein levels only under acute NMDAR blockade conditions. The exact mechanisms and the functional outcome of the interaction between IPR19 and PHB2 in this context remain to be elucidated.

An increase in the PHB2 protein levels under a single dose of MK801 and a significant decrease under MK801 and IPR19 treatment was observed in the cytoplasm and nuclei of cortical neurons, suggesting that PHB2 regulation by both treatments may occur in both cellular compartments. The fact that PHB2 highly correlated with the translocase of outer mitochondrial membrane 20 (TOMM20) protein expression in control and MK801 conditions suggests that PHB2 is primarily localized in the mitochondria under the NMDAR blockade. Apoptosis together with the progressive loss of cortical gray matter have been described in the pathophysiology of SZ [56,57]. PHB2 is ubiquitously expressed, and it has been suggested that it can translocate into the nucleus or mitochondria under apoptotic signals [58]. In the nucleus, PHB2 plays a role as a transcriptional repressor where it directly interacts with the estrogen receptor (ER) and represses ER transcriptional activity [41,59]. In the mitochondria, PHB2 plays a role in many biological functions [30,38], including multiple functions in mitochondria dynamics, mitophagy, stabilization of mitochondrial lipids, and apoptosis [32]. Indeed, PHB2 regulates the cleavage of mitochondrial dynamin such as GTPase (OPA1), which plays a role regulating the inner membrane fusion and cristae structure [60,61]. Moreover, in PHB2 KO mice, the deletion of the m-AAA-protease OMA1 (metalloendopeptidase OMA1) prevents OPA1 destabilization and attenuates neurodegeneration [31]. Recently, PHB2 has been described to regulate stress responses and cell death through OMA1 turnover and cytochrome c release in cortical neurons, providing new insights for the regulation of mitochondrial cell death pathways by prohibitins [61]. All these previous studies point out that an increase in PHB2 levels may lead to a decrease in cytochrome c release and may have a protective role in apoptosis. Since we observe that in the acute models of the disease, there is an increase in the PHB2 protein levels, which is primarily located in the mitochondria, we hypothesize that PHB2 increased levels under the NMDAR blockade may reflect the mitochondrial alterations in SZ; however, whether the increased PHB2 expression is involved in the mitochondrial dysfunction or it is an adaptative change in disturbed mitochondrial function is still unknown. Further studies are needed to investigate how altered mitochondrial-located PHB2 proteins could contribute to the pathophysiology of SZ under NMDAR blockade and IPR19 treatments.

The use of a human postmortem brain constitutes a useful tool to dissect the molecular mechanisms disrupted in psychiatric disorders, but this has limitations. The influence of potential confounding factors must be carefully explored, and we cannot completely exclude their effect in this study, given the sample size of the elderly subjects available. Patients had long-term and heterogenous antipsychotic medications. In this study, no association with patient chlorpromazine doses and PHB2 protein levels in the postmortem human cohort was found, suggesting that the increase in the PHB2 protein levels observed in patients with chronic SZ may not be influenced by long-term antipsychotic medications. Moreover, subchronic treatments in animals and subacute treatments in cells with antipsychotics did not show significantly altered PHB2 protein levels, supporting the fact that the observed increase in the PHB2 protein levels may not be influenced by antipsychotics in chronic stages. Moreover, no differences in the PHB2 protein levels were observed in astrocytes under MK801 treatment compared with those treated with both MK801 and antipsychotics. However, we cannot completely exclude the possible effect of antipsychotic treatments in the PHB2 protein levels in the chronic stages of the disease, and it would be of interest if further studies assessed the possible regulation of PHB2 by antipsychotic treatments in the acute stages of the pathology. Moreover, small variations in the clinical scores up to death may still slightly affect the associations observed; thus, they may need to be taken with caution. Furthermore, since the left DLPFC from SZ patients was paired to the contralateral hemisphere from controls, PHB2 was analyzed in both mice hemispheres. No significant differences were found (Figure S6). Our cohort was constituted only by men and, therefore, further studies with larger cohorts with an equal representation of both genders and if possible, drug-naïve patients would be of interest. In addition, the use of in vivo and in vitro pharmacological inhibitors could also have some limitations. Although mice treated with 0.2 mg/kg MK801 exhibited psychosis-like behavioral phenotypes, motor disturbances have also been described, which are not present in the PCP model followed by a wash out period [62,63]. The selected IPR19 doses in this study were based on a previous study [21]. In addition, in the same study, IPR19 off-targeting experiments, such as the serotonin 5-HT1A receptor, confirmed that the IPR19 improvement in cognitive performance in animal models was not influenced by the off-targets [21]. However, in cells, we cannot rule out if the inhibition of the serotonin 5-HT1A receptor by IPR19 is mediating the modulation of PHB2 protein levels [21]. Further studies are needed to explore this possibility.

## 4. Materials and Methods

### 4.1. Brain Tissue Samples

Postmortem human brain samples from the DLPFC of patients with chronic schizophrenia (SZ) (n = 20) and unaffected subjects (C) (n = 20) with no history of psychiatric episodes were obtained from the collection of Parc Sanitari Sant Joan de Déu [64] and the Institute of Neuropathology Brain Bank (HUB-ICO-IDIBELL Biobank), following the guidelines of Spanish legislation and the approval of the local ethics committees. The study was approved by the Institutional Ethics Committee of Parc Sanitari Sant Joan de Déu. All deaths were due to natural causes. Neuropathologists from HUB-ICO-IDIBELL Biobank examined the contralateral hemisphere for signs of neurodegenerative disorders in both SZ and unaffected subjects. Written informed consent was obtained for each subject. Experienced clinical examiners interviewed each donor antemortem to confirm SZ diagnosis according to the Diagnostic and Statistical Manual of Mental Disorders IV (DSM-IV) and International Classification of Diseases 10 (ICD-10) criteria. Patients and unaffected individuals were selected based on the following criteria: negative medical records on the presence of neurological disorders or drug abuse, and accidental or natural cause of death that does not compromise the integrity of the region of interest and brain pH higher than 6. The demographic-, clinical-, and tissue-related characteristics of this cohort are provided in Table 1 and Table S1. SZ patients were institutionalized donors with long duration of the illness and prominent negative symptoms evaluated with Positive and Negative Syndrome Scale (PANSS) (m.d. 31.94 ± 10.10) and the Clinical Global Impression–Schizophrenia scale (CGI-SCH) (m.d. 5.56 ± 0.98). We matched SZ and unaffected subjects by age, post-mortem delay, and pH. The last mean daily chlorpromazine equivalent dose for the antipsychotic treatment of patients was calculated based on the electronic records of last drug prescriptions administered up to death, as described previously [65] (Table 1). Specimens of the DLPFC (Brodmann area 9) were dissected, extending from the pial surface to white matter and only including gray matter and they were immediately stored at −80 °C. Due to collection methods in each institution, left dorsolateral prefrontal cortex from SZ patients was paired with the contralateral hemisphere from controls.

Models of psychosis-like behavioral phenotype [62,66] were prepared from eight-week-old C57BL/6J mice (Charles River, Wilmington, MA, USA), as previously described [46] (n = 6 per group). Animals were treated with a single intraperitoneal injection of (+)-5-methyl-10,11-dihydro-5H-dibenzo[a,d]-cyclohepten-5,10-imine hydrogen maleate (MK801) (0.2 mg/kg; Sigma-Aldrich, Saint Louis, MO, USA), IPR19 (5 mg/kg) or with vehicle (phosphate-buffered saline). Mice were killed by cervical dislocation 35 min after the injection and mice brains were rapidly removed. As a subchronic animal model of SZ [63,67], C57/BL6J mice animals aged between 8 and 10 weeks were treated as previously described in the work by Prades and colleagues [21]. Animals were treated with vehicle or phencyclidine (PCP) (10 mg/kg; once a day on days 1–5 and 8–12) in saline for 10 days. Experiments were conducted after a five-day washout period. Thirty-five minutes before prefrontal cortex dissection, animals were injected i.p. with 5 mg/kg of IPR19 or vehicle (VEH n = 5, PCP, and PCP and IPR19 groups n = 8).

For pharmacological treatments with antipsychotic drugs, 200–250 g Sprague Dawley rats (Harlan Laboratories, Indianapolis, IN, USA) were treated as previously described [45] (n = 6, per group). Animals were treated with a daily intraperitoneal injection of haloperidol (0.5 mg/kg/day; Sigma Aldrich), clozapine (20 mg/kg/day; Sigma Aldrich), or vehicle (phosphate-buffered saline) for 21 days (n = 6). Rats were sacrificed at day 22 by decapitation.

For tissue preparation of left and right brain hemispheres of mice, six-month-old CD1 (ICR) mice (Charles River) were prepared as previously described [45] (n = 5 per group). Animals were anesthetized with 2-chloro-2-(difluormethoxy)-1,1,1-trifuloro-ethane (isoflurane; Abbvie, North Chicago, IL, USA) and killed by decapitation.All animals were randomly assigned to one of the treatment groups and maintained on a 12 h light/dark cycle with access to food and water ad libitum. Animal experimentation protocols were approved by the Institutional Animal Care and Use Committees of the University of Barcelona, the University of Basque Country and the University of Cantabria. All experimental procedures were conducted in accordance with the Declaration of Helsinki, the Spanish legislation, and the European Communities Council Directive on “Protection of Animals Used in Experimental and Other Scientific Purposes” (86/609/EEC).

### 4.2. Rat Cortical Cultures

Cerebral cortical astrocytes cultures were prepared from Sprague Dawley postnatal (P1) rat pups (Harlan Laboratories, Indianapolis, IN, USA), as previously described [45]. Briefly, the dissociated cells were plated into 150 mm dishes (8 × 10^6^ viable cells/dish) in DMEM, 1% glutamine, and 1% penicillin/streptomycin (Sigma-Aldrich Saint Louis, MO, USA). The medium was replaced 2 h after plating to remove non-adherent cells and every 2–3 days thereafter. After that, cells were plated into 6-well plates (2 × 10^5^ cells/well) for Western blot analysis.

Prefrontal cortical cell neurons cultures were prepared from embryonic (E17) Sprague Dawley rat pups. The dissociated cells were plated into 6-well plates previously treated with poly-D-lysine (Sigma) (5 × 10^4^ viable cells/dish) in DMEM (Life technologies, Carlsbad, CA, USA), 10% Fetal Bobine Serum (Sigma-Aldrich, Saint Louis, MO, USA), and 1% penicillin/streptomycin (Life technologies, Carlsbad, CA, USA). The medium was replaced with Neurobasal with 2% B27, 1% Glutamax, and 0.5% penicillin/streptomycin (Life technologies) 2 h after plating to remove non-adherent cells and every 2–3 days thereafter. For acute NMDAR blockade experiments, 10 μM MK801 doses were used. For the subacute NMDAR blockade experiments, cells were treated with 50 µM MK801 during 72 h as previously described [68].Cells were also treated with 50 µM IPR19. Since the patient cohort was chronically treated with antipsychotics, cells were treated with 10 μM clozapine or 1 μM haloperidol (Sigma Aldrich, Saint Louis, MO, USA) only in subchronic conditions (72 h), and not under acute treatments (1 h) where antipsychotics do not have a short-time expected effect. Protein extracts were analyzed by immunoblot.

### 4.3. Protein Extraction

All brain samples were homogenized at 4 °C in a Bullet Blender (Next Advance) with 3.2 mm stainless steel beads (Next Advance, Troy, NY, USA) for 2 min with lysis buffer. Protein extracts were incubated on ice for 30 min, sonicated for 8 s at 21% amplitude, and centrifuged at 18,200× g for 15 min at 4 °C. Protein concentration was determined using the Bradford assay (BioRad, Hercules, CA, USA).

### 4.4. Immunoblotting

Protein lysates from human tissue (50 µg), rodent tissue (20 µg), and cell cultures (20 µg) were resolved by SDS/PAGE electrophoresis and immunoblotted with the primary antibodies: PHB2 (Abcam, Cambridge, UK), α-TUBULIN (Sigma-Aldrich), β-ACTIN (Sigma-Aldrich), and glyceraldehydes-3-phosphate dehydrogenase (GAPDH, Millipore, Burlington, MA, USA). Densitometric quantification of PHB2 protein was performed using Quantity One software version 4.1.1 (BioRad, Hercules, CA, USA) in duplicate samples. In human postmortem brain tissue immunoblots, PHB2 protein levels were referred to as the geometric mean of the following housekeeping proteins: TUBULIN, ACTIN, and GAPDH. PHB2 levels were also referred to as the levels of a control sample.

### 4.5. Reverse Transcription Quantitative PCR (RT-qPCR)

Total RNA was extracted using Trizol reagent (Sigma-Aldrich, Saint Louis, MO, USA). We controlled RNA quality by assessing RNA integrity number for each sample with an Agilent Bioanalyzer (Agilent Technologies, Santa Clara, CA, USA). First strand cDNA was synthesized from 2 µg of RNA using SuperScript III (Invitrogen, Waltham, MA, USA). Applied Biosystems Taqman master mix formulation for gene expression, probe, and primers was used for quantitative real-time PCR. Assay identification primers for target genes were as follows: Hs00200720_m1 for PHB2; Hs00427620_m1 for TATA-binding protein (TBP), and 4326320E for beta-glucuronidase (GUSB) genes (Applied Biosystems, Waltham, MA, USA). Amplification of selected cDNA samples was carried out on an Applied Biosystems model 7500 Real-Time PCR system. The relative quantification of the gene expression levels in each sample was performed using the comparative cycle threshold, ΔΔCT method, and validated for PHB2 gene (Figure S7). PHB2 mRNA expression levels were normalized to a reference sample and the geometrical mean of TBP and GUSB.

### 4.6. Immunocytochemistry and Confocal Imaging

The immunocytochemistry was performed from cortical neurons of frontal pole prepared from embryonic (E17) Sprague Dawley rat pups. The dissociated cells were seeded onto glass coverslips previously treated with poly-D-lysine (Sigma-Aldrich, Saint Louis, MO, USA) (5 × 10^4^ viable cells/well). Cells were fixed with 4% paraformaldehyde for 20 min at room temperature followed by permeabilization with 0.2% Triton X-100 in TBS. Neurons were incubated with blocking solution for 1 h and immunostained with antibodies against PHB2 (abcam, Cambridge, UK) and TOMM20 (abcam Cambridge, UK). For secondary antibodies, we used goat-anti-rabbit Alexa Fluor 594 (Invitrogen, Waltham, MA, USA) and goat-anti-mouse Dye Light 488 (Invitrogen, Waltham, MA, USA) at room temperature for 1 h. Nuclei were visualized with DAPI stain (Invitrogen, Waltham, MA, USA). Negative control image for secondary antibodies is shown in Figure S8.

Confocal microscopy acquisition was performed with Leica TCS SP8 STED 3X equipped with white light laser confocal microscope with hybrid detectors (Leica Microsystems, Mannheim, Germany). Confocal microscopy quantification acquisition parameters are described in the Supplementary Table S3. Cells were excited sequentially at three different wavelengths: 405 nm, 488 nm, and 555 nm, which, respectively, excited DAPI, TOMM20, and PHB2. Fluorescence intensities images of an average of fifteen randomly selected microscopic fields of cells that were semi-quantitatively analyzed by densitometry (Image J software (NIH) software version 1.53q). All images were acquired at 63× magnification with 1.4 numerical aperture objective, digitized into format of 1024 × 1024 pixels and 12-bit depth. Ten stacks every 0.5 μm along with the cell thickness were acquired in Z axis. ImageJ (NIH) software version 1.53q was used to analyze fluorescent intensities of proteins. Regions of interest were used for the quantification of nuclear and cytoplasm signals. Image segmentation of the blue channel was used to delimitate nuclei regions and applied to the red channel to analyze those pixels in the nuclei region. One-hundred cells were quantified in each condition in three independent experiments. For the co-localization analysis, the JACoP plugin (Just Another Colocalization Plugin) in Image J (NIH) software version 1.53q was used and Pearson’s correlation was used to assess the degree of co-localization between PHB2 and TOMM20.

### 4.7. Statistical Analysis

Normal distribution of the variables was carried out by D’Agostino and Pearson omnibus normality test. Grubbs test and Pierce test were used to detect outliers. Fisher exact test (qualitative variables), Student t-test (parametric quantitative variables), and Mann–Whitney U test (non-parametric quantitative variables) were used to compare demographic- and tissue-related features between control and SZ groups. Two-tailed tests were used and significance level was set to 0.05.

Bivariate analyses were carried out to detect association of our variables with potential confounding factors (age, postmortem delay, pH, RIN, daily antipsychotic dose, age of onset, and duration of the illness) and correlation with the different scale scores (PANSS and FAB), using Spearman or Pearson correlations for non-parametric and for parametric variables, respectively. When comparing more than two groups, a statistical analysis was performed using ANOVA followed by Bonferroni’s (cell culture) or two-stage step-up method for Benjamini, Kriger, and Yekutieli (animals) post hoc comparisons, as indicated by post-host test in each figure. Co-localization analysis was performed using JACoP plug-in on ImageJ (NIH) software version 1.53q. Pearson’s correlation coefficient (r) was used to quantify colocalization [69]. Statistical analysis was performed with GraphPad Prism version 5.00 and SPSS (IBM)23.

## 5. Conclusions

In conclusion, our findings indicate that PHB2 protein levels may be altered in the postmortem prefrontal cortex of schizophrenia patients and are associated with the cognitive impairments described in the pathology. Moreover, our results suggest that new cognitive therapeutic strategies could modulate PHB2 protein levels and may help to counteract cognitive impairments in SZ.

Furthermore, our findings suggest that PHB2 may be involved in the mechanisms deregulated by altered glutamate signaling pathways in the initial stages of the disorder and may contribute to the development of cognitive symptoms linked to the NMDAR hypoactivity state in schizophrenia. Indeed, our results suggest that IPR19 may restore the upregulation of PHB2 in acute NMDAR hypoactivity and in both cortical neurons and astrocytes, indicating that the modulation of PHB2 could compensate for NMDAR-dependent cognitive impairments in SZ.

## Figures and Tables

**Table 1:** Demographic-, clinical-, and tissue-related features of cases in the whole cohort (n = 40).

	Schizophrenia (n = 20)	Controls (n = 20)	Statistic	*p* Value
Gender (male)	100% (n = 20)	100% (n = 20)	N/A	1.000 ^a^
Age (years)	69 ± 11	74 ± 10	1.35; 38 ^b^	0.184
PMD (hours)	4.71 ± 2.51	5.45 ± 1.72	1.47; 38 ^b^	0.150
pH	6.74 ± 0.52	6.76 ± 0.35	195.5 ^c^	0.912
SZ diagnosis		N/A	N/A	N/A
Chronic residual	70% (n = 14)			
Chronic paranoid	10% (n = 2)			
Chronic disorganized	10% (n = 2)			
Chronic catatonic	5% (n = 1)			
Simple	5% (n = 1)			
Age of onset of illness (years)	22 ± 7	N/A	N/A	N/A
Duration of illness (years)	51 ± 10	N/A	N/A	N/A
Dosage of AP (mg/day) ^d^	584.60 ± 518.65	N/A	N/A	N/A
AP treatment		N/A	N/A	N/A
First-generation AP	30% (n = 6)			
Second-generation AP	50% (n = 10)			
None	20% (n = 4)			

Mean ± standard deviation or relative frequency are shown for each variable; PMD, postmortem delay; SZ, schizophrenia; AP, antipsychotic; N/A, not applicable. ^a^ Fisher’s exact test is shown for categorical variables. ^b^ T statistic and degrees of freedom are shown for parametric variables. ^c^ Mann–Whitney U is shown for non-parametric variables. ^d^ Last chlorpromazine equivalent dose was calculated based on the electronic records of drug prescriptions of the patients.

## Data Availability

The data presented in this study are available on request from the corresponding author.

## Supplementary Materials

The following supporting information can be downloaded at: https://www.mdpi.com/article/10.3390/ijms24076016/s1.
