# Supplementary material for: Inhibition of Prolyl Oligopeptidase Restores Prohibitin 2 Levels in Psychosis Models: Relationship to Cognitive Deficits in Schizophrenia
Source: Int J Mol Sci. 2023 Mar 23;24(7):6016. doi: 10.3390/ijms24076016 (PMC10093989; doi:10.3390/ijms24076016)
Supplement: Supplementary file 1 [file ijms-24-06016-s001.zip › ijms-2098311-supplementary.pdf]

SUPPLEMENTARY MATERIAL

Figure S1

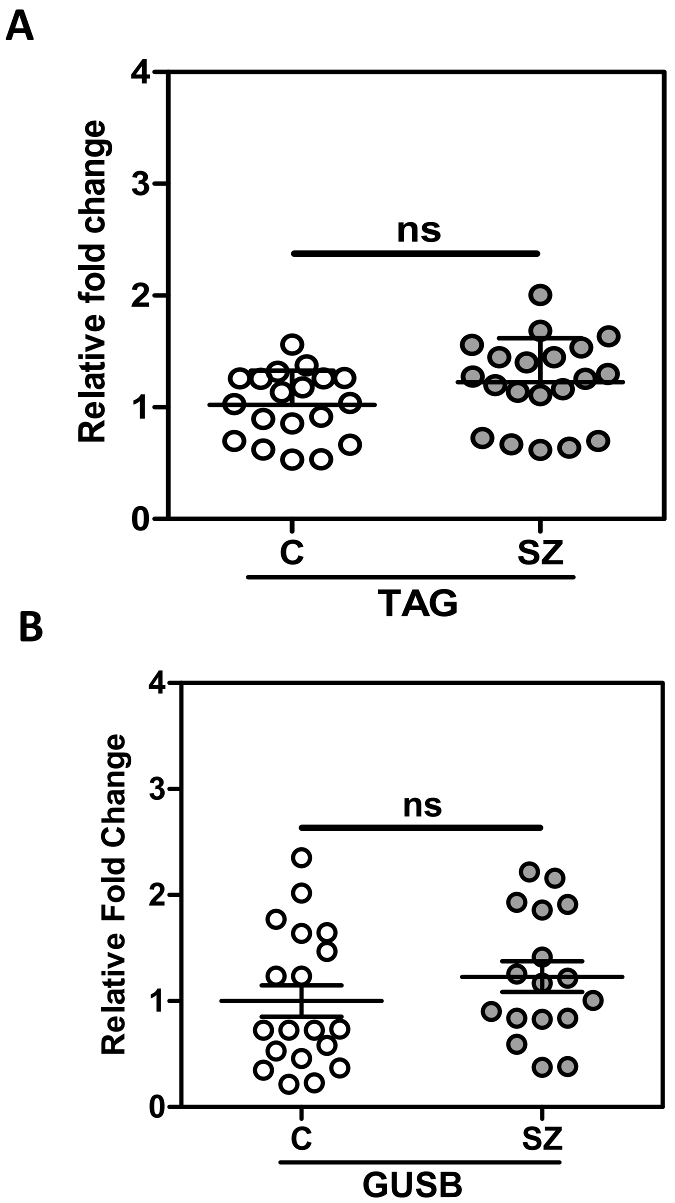

Figure S1. The expression of the house-keeping (HK) genes and proteins is not significantly altered in the postmortem brain of schizophrenia subjects. (A) The three-reference protein, α-Tubulin (T), β-Actin (A) and GAPDH (G) were determined by immunoblot in prefrontal cortex samples from control individuals (n=20) and schizophrenia patients (n=20). The graph represents the geometrical mean of the HK proteins. Statistical analysis was performed using Student's t-test (n.s., not significant). **(B)** Beta glucuronidase (GUSB) levels from the prefrontal cortex of control individuals (C=19) and schizophrenia patients (SZ=17) were determined by RT-qPCR. Values represent the mean ± standard error of the mean for each group. Mean fold-change values of each group were compared with Student's t-test (n.s, not significant).

Figure S2

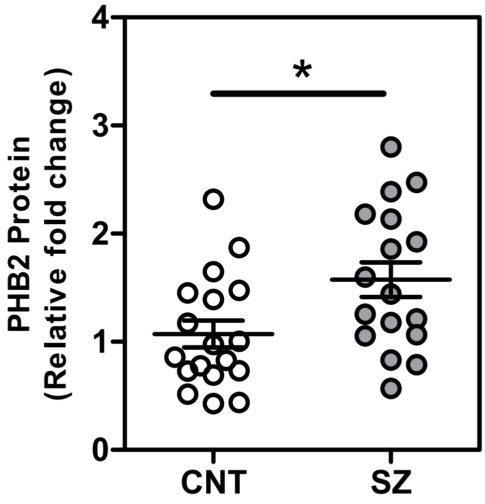

Figure S2: Increased PHB2 protein levels in the subgroup of schizophrenia patients. Differences between PHB2 protein levels from the prefrontal cortex of control individuals and schizophrenia patients in the cohort used for PHB2 mRNA analysis were maintained. Values represent the mean $\pm$ standard error of the mean for each group. Mean fold-change values of each group were compared with Student's t-test (*p<0.01).

Figure S3

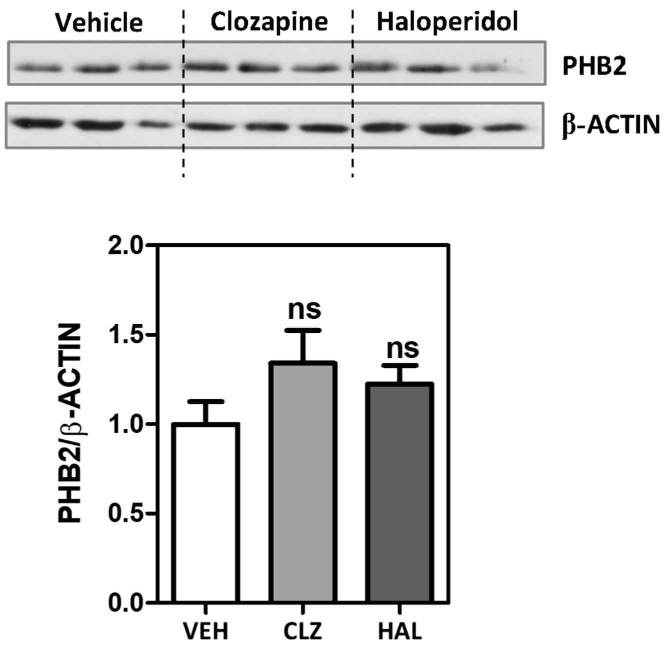

Figure S3: No differences in PHB2 protein levels under clozapine and haloperidol treatments.

PHB2 protein levels from the frontal cortex of Sprague Dawley rats treated with vehicle (VEH), 20 mg/kg/day clozapine (CLZ) or 0.5 mg/kg/day haloperidol (HAL) for 3 weeks were determined by immunoblot (n=6, per group). Representative Immunoblot images for PHB2 and β-actin in untreated and treated animals are shown. Each bar represents the mean and the standard error of the mean for each group for PHB2 and β-actin protein levels of at least two independent analyses. Protein levels of PHB2 were normalized to the β-actin values and referred to the vehicle treated animals. Statistical analysis was performed using ANOVA followed by Bonferroni's post hoc comparison between untreated condition and different treatments (ANOVA: $F[2.15]=1.494$; $p = 0.256$); (ns, not significant). Original western blot images are provided in supplementary information (I).

Figure S4

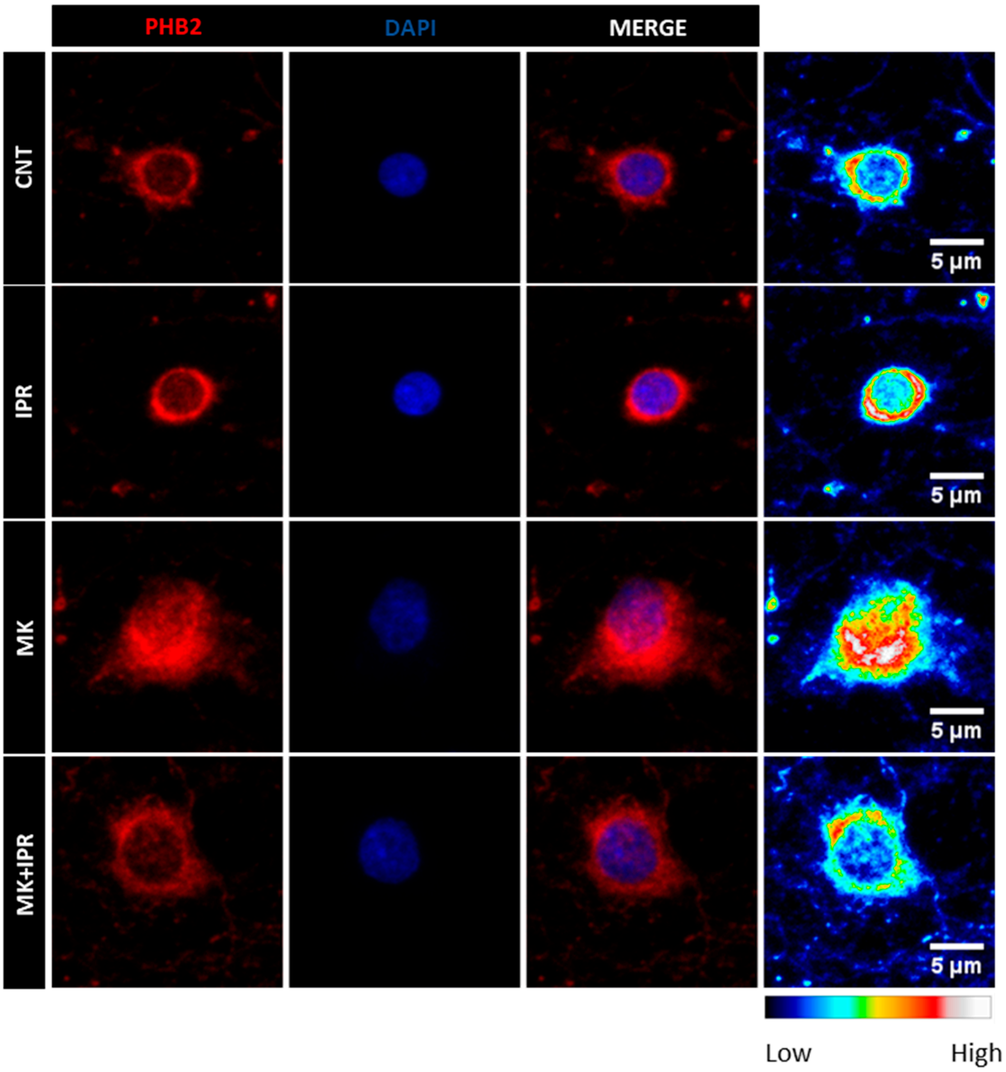

Figure S4. Immunocytochemistry of PHB2 in cytoplasm and nucleus of cortical neurons. Panel shows representative images of cortical neurons stained with PHB2 (1:50) and DAPI (1:400) treated with vehicle (DMSO), N-methyl-D-aspartate receptor antagonist MK801 (MK 10µM) and the prolyl oligopeptidase (IPR19 50µM) used in the region of interest analysis to determine PHB2 intensity in the cytoplasm and nucleus.

Figure S5

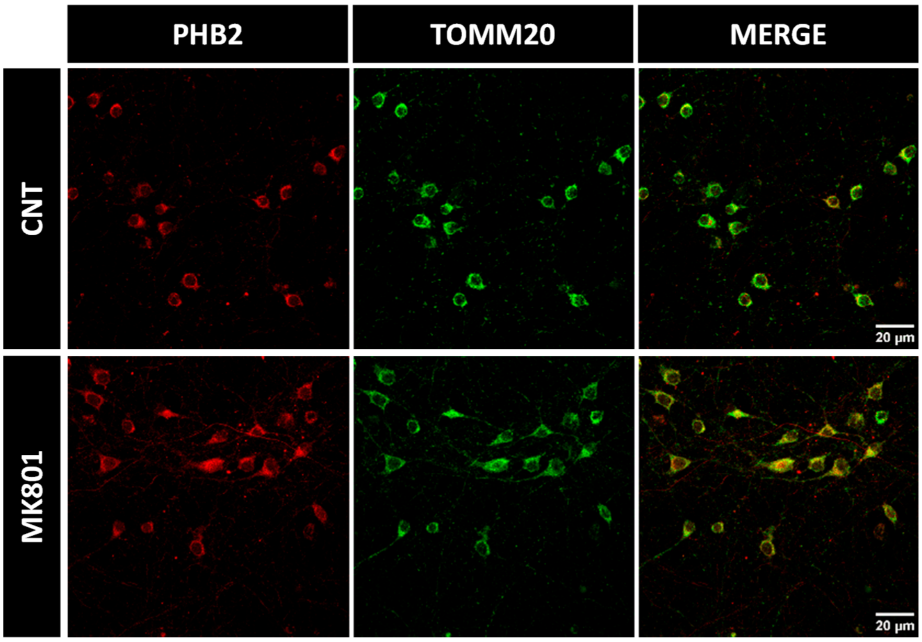

Figure S5. Immunocytochemistry of PHB2 in cortical neurons. Panel shows representative images of the immunostaining of PHB2 (1:50), DAPI (1:400), TOMM20 (1:100) in cortical neurons treated with vehicle (DMSO) or N-methyl-D-aspartate receptor antagonist MK801 (MK 10μM). Merge images show the interaction between TOMM20 and PHB2 proteins (yellow).

Figure S6

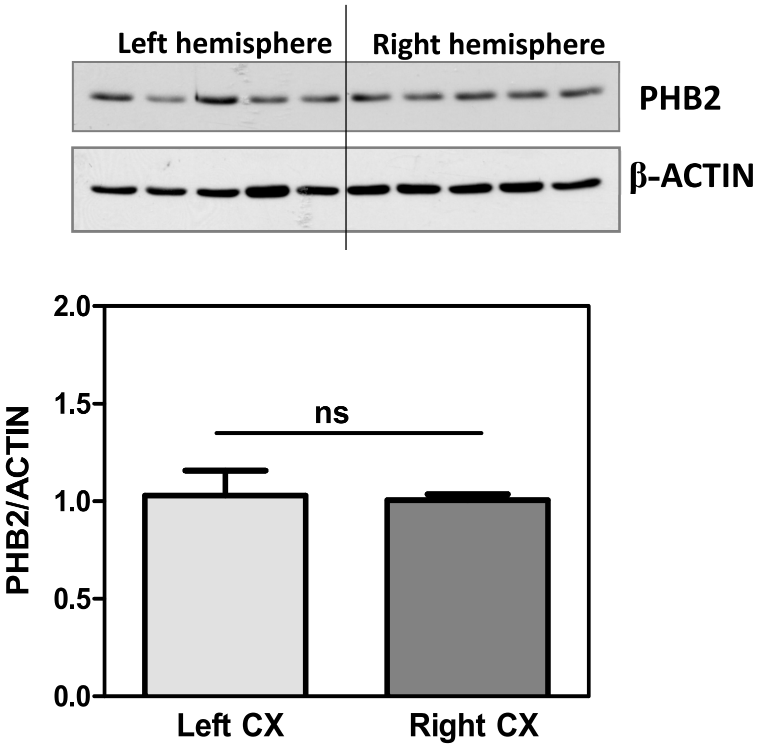

Figure S6. PHB2 protein levels do not significantly differ between mouse brain hemispheres. PHB2 protein levels were determined by immunoblotting in protein extracts from the left and right hemispheres of 6-month-old CD1 mice (n=5, per group). PHB2 protein levels were normalised to β-actin values. Images show representative PHB2 and β-ACTIN immunoblots. Statistical analysis was performed using two-tailed unpaired t test (ns, not significant). Original western blot images are provided in supplementary information (J).

Figure S7

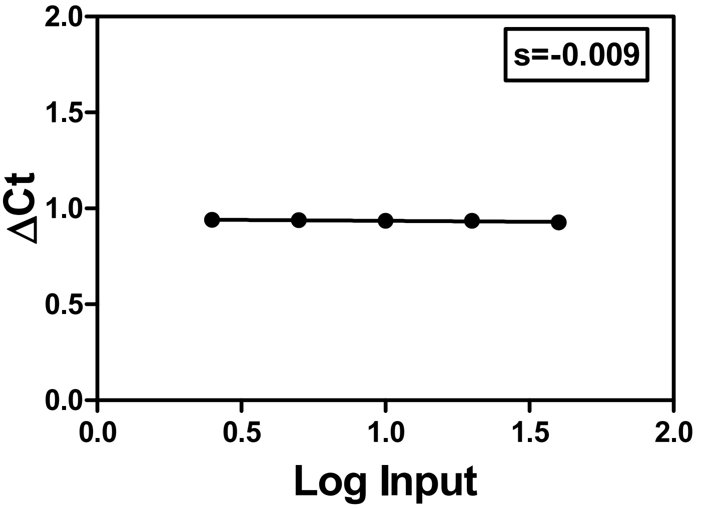

Figure S7. ΔΔCt validation method. PHB2 mRNA levels were analysed by qRT-PCR in prefrontal cortex in a serial dilution of a pool of control samples. PHB2 mRNA levels were normalized to GUSB levels and values were adjusted to a linear regression. The figure shows the slope of the adjusted line which is below 0.1 and therefore, passed the validation test.

Figure S8

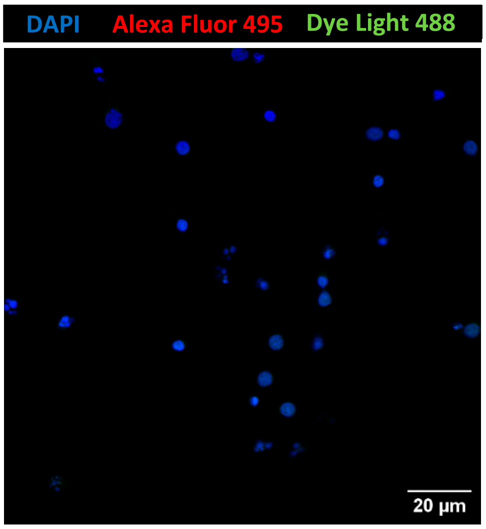

Figure S8. Negative control for secondary antibodies. Negative control for secondary antibodies goat anti-mouse Dye light 488 and goat anti-rabbit Alexa Fluor 594. All negative controls were used without incubating cortical neurons with the corresponding primary antibody.

Table S1. Demographic and clinical tissue related features of cases in a subgroup of patients for mRNA analysis (n=36).

	Schizophrenia (n=17)	Controls (n=19)	Statistic	p value
Gender (male)	100% (n=17)	100% (n=19)	N/A	1.000[a]
Age (years)	74 ± 10	70 ± 11	0.89; 26[b]	0.384
PMD (hours)	4.62 ± 2.55	5.74 ± 1.67	1.44; 26[b]	0.160
pH	6.76 ± 0.29	6.76 ± 0.43	93.00[c]	0.849
RIN	7.11 ± 0.76	7.66 ± 0.60	1.91; 25[b]	0.068
SZ diagnosis		N/A	N/A	N/A
Chronic residual	70% (n=12)			
Chronic paranoid	12% (n=2)			
Chronic disorganized	6% (n=1)			
Chronic catatonic	6% (n=1)			
Simple	6%(n=1)			
Age of onset of illness (years)	23 ± 7	N/A	N/A	N/A
Duration of illness (years)	51 ± 9	N/A	N/A	N/A
Dosage of AP (mg/day)[d]	612.8 ± 544	N/A	N/A	N/A
AP treatment		N/A	N/A	N/A
First-generation AP	33% (n=5)			
Second-generation AP	53% (n=8)			
None	13% (n=2)			

Mean ± standard deviation or relative frequency are shown for each variable; PMD, *postmortem* delay; RIN, RNA integrity Number; SZ, schizophrenia; AP, antipsychotic; N/A, not applicable.

[a] Fisher's exact test is shown for categorical variables

[b] T-statistic and degrees of freedom are shown for parametric variables.

[c] Mann-Whitney U is shown for non-parametric variables

[d] Last chlorpromazine equivalent dose was calculated based on the electronic records of drug prescriptions of the patients.

Table S2. Association analysis of other variables in the validation cohort.

	PHB2 protein	
Correlation	SZ-C (n=40) †	SZ (n=20)
Age	0.324 [b]	0.342 [a]
PMD	0.096 [b]	0.136 [a]
pH	0.134 [b]	0.017 [a]
Age of onset	N/A	0.064 [a]
Daily AP dose	N/A	-0.351 [a]
Duration of the illness	N/A	0.266 [b]

PMD, postmortem delay; AP, antipsychotic; N/A, not applicable. [a] r, Pearson's r correlation; [b] r', Spearman's r correlation. †An outlier was detected for PHB2 protein values by the Grubbs test and therefore excluded from the analysis (PHB2: control group, n=19, schizophrenia group, n=20). Significant associations are indicated in bold ($p<0.05$).

Table S3. Confocal microscopy quantification acquisition parameters.

**Imaging format**	1024 x1024 pixels
**Sections number (3D)**	10
**Step Z (um)**	0.5
**Dynamic Range**	12 bits
**Speed**	600 Hz (Bidirectionality)
**Objective**	HCPLAPO CS2 63X/1.40 OIL
**Zoom**	1,25
**Pinhole Airy**	1
**Excitation wavelength and AOTF (Channel 1)**	405 nm (0.6%)
**Excitation wavelength and AOTF (Channel 2)**	488 nm (18.5%)
**Excitation wavelength and AOTF (Channel 3)**	594 nm (29.9%)
**Range emission Channel 1**	420-465 nm
**Range emission Channel 2**	512-556 nm
**Range emission Channel 3**	606-760 nm
**Gain Channel 1**	11%
**Gain Channel 2**	10%
**Gain Channel 3**	30%

Descriptive table of the acquisition parameters used in the quantification experiments which have been kept constant throughout the experimentation.
